# Regulation of transcription by eukaryotic-like serine-threonine kinases and phosphatases in Gram-positive bacterial pathogens 

**DOI:** 10.4161/21505594.2014.983404

**Published:** 2015-01-20

**Authors:** David P Wright, Andrew T Ulijasz

**Affiliations:** MRC Centre for Molecular Bacteriology and Infection (CMBI); Imperial College London; London, UK

**Keywords:** bacteria, infection, phosphorylation, serine threonine kinase, serine threonine phosphatase, transcription

## Abstract

Bacterial eukaryotic-like serine threonine kinases (eSTKs) and serine threonine phosphatases (eSTPs) have emerged as important signaling elements that are indispensable for pathogenesis. Differing considerably from their histidine kinase counterparts, few eSTK genes are encoded within the average bacterial genome, and their targets are pleiotropic in nature instead of exclusive. The growing list of important eSTK/P substrates includes proteins involved in translation, cell division, peptidoglycan synthesis, antibiotic tolerance, resistance to innate immunity and control of virulence factors. Recently it has come to light that eSTK/Ps also directly modulate transcriptional machinery in many microbial pathogens. This novel form of regulation is now emerging as an additional means by which bacteria can alter their transcriptomes in response to host-specific environmental stimuli. Here we focus on the ability of eSTKs and eSTPs in Gram-positive bacterial pathogens to directly modulate transcription, the known mechanistic outcomes of these modifications, and their roles as an added layer of complexity in controlling targeted RNA synthesis to enhance virulence potential.

## Abbreviations

TCS: two-component signaling
OCS: one-component signaling
eSTK: eukaryotic-like serine-threonine kinase
eSTP: eukaryotic-like serine-threonine phosphatase
PASTA: penicillin-binding protein and Ser/Thr kinase associated
REC: receiver
wHTH: winged helix-turn-helix
PTM: posttranslational modification
PPM: protein phosphatase metal binding
ROS: reactive oxygen species

## Introduction

### Eukaryotic-like serine-threonine kinases (eSTKs) and phosphatases (eSTPs) in bacterial pathogens

To allow their continued survival pathogenic microorganisms have evolved multiple and sophisticated mechanisms to adapt to environmental changes within the host. These mechanisms probe the extracellular environment for variations in, for example, nutrient concentrations, oxygen tension and other perceivable dangers to enable them to elicit the appropriate physiological alterations in response. Extracellular signals are commonly relayed into the cells by means of reversible phosphorylation through protein kinases and phosphatases, and in doing so modulate their target activities. The most well described prokaryotic signaling cascades are the two-component signaling (TCS) systems.^1,2^ The typical TCS system makes use of a membrane bound sensory histidine kinase capable of detecting extracellular signals and when activated, autophosphorylates a conserved histidine residue within the ATPase catalytic domain of the protein. In the most basic and common system, the phosphoryl group is then transferred onto a conserved aspartate contained within the RECiever (or REC domain) of a single cognate effector, often a response regulator transcription factor.^2^ This event then triggers the activation of the C-terminal effector domain portion of the protein, the majority of which are DNA-binding domains, to result in subsequent interaction and regulation of specific promoters to facilitate the microbe's timely environmental adaptation.^3^

**Figure 1.**
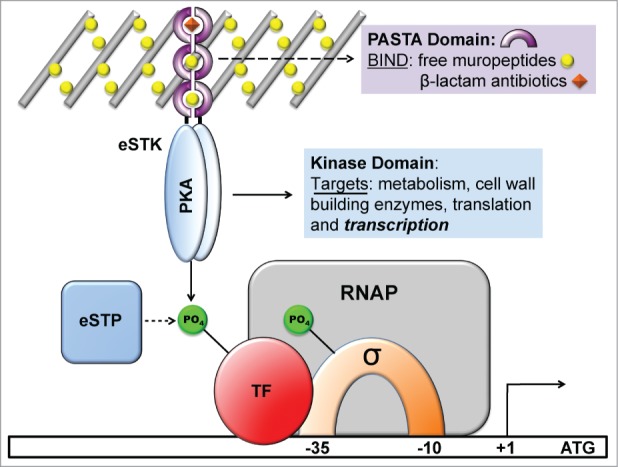
Schematic diagram showing the functional roles of eukaryotic serine-threonine kinases (eSTKs) and phosphatases (eSTPs). eSTKs contain an N-terminal cyclic AMP (cAMP)-dependent Protein Kinase domain (PKA; colored blue) and usually a C-terminal sensory domain. In non-mycobacterial Gram-positive bacteria the sensory domain is often a PASTA domain (purple half-rings). PASTA domains have been shown to bind free muropeptides (yellow balls) and cell wall-targeting antibiotics (*e.g.* β-lactams, orange diamonds). Once activated, eSTKs will dimerize and their intracellular kinase domains phosphorylate pleiotropic targets to produce specific cellular responses. eSTPs can, in turn, remove the phosphate(s) from an eSTK or substrates. Targets include metabolic enzymes, cell wall building and cell division regulatory enzymes such as DivIVA or FtsZ, translational machinery, and transcription factors (TFs) including sigma factors (σ) and possibly RNA polymerase (RNAP) holoenzyme subunits.

Although TCS has traditionally enjoyed the ‘lion's share’ of attention in the bacterial signaling field and were once thought the only means by which microbes could sense their environment, it has more recently come to light that eSTKs and eSTPs also play important roles in prokaryotic adaptation. Unlike their TCS counterparts, eSTK/Ps are usually present at far fewer numbers per bacterial genome, with only one cognate pair found in many Gram-positive species. eSTKs were initially identified in the soil-dwelling, Gram-negative microbe *Myxococcus xanthus* for their involvement in the normal development of this organism.^4^ However, most subsequent studies have focused on these signaling cascades as being indispensable for virulence in a variety of human pathogens.^5-15^ The ‘genomic age’ has revealed a considerable expansion of the eSTK protein family and the presence of their cognate eSTPs in most prokaryotic Phylums, which include important Gram-negative human pathogens such as *Pseudomonas aeruginosa*,^9,^^16^ and many Gram-positives such as *Enterococcus faecalis*,^17^ *Staphylococcus aureus,*^12,14,^^18-20^ *Mycobacterium tuberculosis*,^6,21,22^ *Streptococcus pneumoniae*,^7,23,24^ *Streptococcus agalactiae*^8^ and *Streptococcus pyogenes*.^10,^^25^ This discovery led to the realization that unlike TCS histidine kinases, which almost exclusively target a single response regulator partner, eSTK substrates are instead quite pleiotropic in nature. This feature enables a single eSTK/P pair to regulate a large variety of proteins crucial for important cellular functions, including transcription and translation, cell division, dormancy, antibiotic resistance, bacterial persistence and expression of virulence factors such as exotoxins^20^ (**Fig. 1**). The diversity of eSTK substrates is highlighted by several proteomic studies. Some examples include one from *M. tuberculosis* where 301 protein substrates were identified for the unusually high 11 eSTKs found in this organism,^22,26^ another from *Listeria monocytogenes* where 62 interacting partners were identified for its 2 eSTKs,^27^ and a study conducted in *S. agalactiae* where 10 potential substrates were discovered for its single known eSTK.^28^ However, at present many identified posttranslational modifications (PTMs) associated with regulatory roles in virulence and other important bacterial functions have only been verified by *in vitro* experimentation, and thus require more difficult genetic and biochemical assays to demonstrate their relevance *in vivo*. **Table 1** lists the eSTK transcription modulating targets described in this review and highlights these issues. For a more comprehensive list of eSTKs and their substrates see reference 29.

**Table 1.** Gram-positive eSTK-regulated transcription factors involved in virulence and infection^c^

Sp.	Name	Family	Function	eSTK	Phospho-residue(s)	Domain	Effect on DNA binding	*In vitro* or *In vivo*	^b^Ref.
*S. aureus*	SarA	SarA/ MgrA	Virulence	Stk1 (PknB), SA0077 (Stk2)	Thr(s)/ Ser(s)/C9	DD	Increase/ Decrease	*In vitro, in vivo*	^84,88^
	SarZ	SarA/ MgrA	Redox	Stk1 (PknB)	C13	DD	Decrease	*In vitro, in vivo*	^88^
	MgrA	SarA/ MgrA	Antibiotic resistance	Stk1 (PknB)	S110, S113, C11	DD	Decrease	*In vitro, in vivo*	^82,88^
	CymR	SarA/ MgrA	Cysteine metabolism	Stk1 (PknB)	C25	DD	Unknown	*In vitro, in vivo*	^88^
	GraR	TCS	Antibiotic resistance	Stk1 (PknB)	T128, T130, T149	DBD	Increase	*In vitro*	^102^
	CcpA	LysR	Central metabolism/virulence	Stk1 (PknB)	T18, T33	DBD	Decrease	In vitro, in vivo*	^97^
	VraR	NarL	Antibiotic resistance	Stk1 (PknB)	T106, T119, T175 T178	REC, DBD	Decrease	In vitro, in vivo*	^103^
*M. tuberculosis*	VirS	AraC	Stress response	PknK	Unknown	Unknown	Unknown	In vitro, in vivo*	^129^
	DosR	TCS-like	Redox, Dormancy	PknH	T198, T205	REC	Increase	*In vitro*	^108^
	EmbR	SARP	Cell wall modification	PknA, PknB, PknH	Thr(s), T209	FHA, BTA	Increase	*In vitro, in vivo*	^112,118,119^
	Rv0681	TetR	Unknown	PknH	T35	DBD	Unknown	*In vitro*	^119^
	EthR	TetR	Antibiotic resistance	PknF	T2, T3, S4, S7	Un-structured	Increase	*In vitro*	^122^
	Rv2175c	TCS	Cell envelope	PknL	T9	Un-structured	Unaffected	*In vitro*	^125^
	RshA	anti-σ factor	Stress response	PknB	T94	Unknown	Unknown	*In vitro, in vivo*	^131^
	SigH	σ factor	Stress response	PknB	T26, T106	Unknown	Unknown	*In vitro, in vivo*	^131^
	Rv0516c	anti-anti-σ factor	Unknown	PknD	T2	Unknown	Unknown	*In vitro, in vivo*	^132^
*GBS*	CovR	OmpR/ PhoB	Virulence	Stk1	T65	REC	Decrease	*In vitro, in vivo*	^138,139^
*GAS*	WalR	OmpR/ PhoB	Cell wall, division	SP-STK	Thr(s)	Unknown	Unknown	*In vitro, in vivo*	^10^
	CovR	OmpR/ PhoB	Virulence	SP-STK	T65	Unknown	Increase	*In vitro, in vivo*	^139^
*S. pneumoniae*	RitR	OmpR/ PhoB	Iron uptake, ROS stress	StkP	Unknown	DBD	Unknown	*In vitro*	^24^
	RR06	OmpR/ PhoB	Adhesion	StkP	Thr(s)	Unknown	Increase	*In vitro, in vivo*	^149^

a DBD, DNA-Binding Domain; REC, Receiver Domain; DD, Dimerization Domain; σ, Sigma Factor; TCS, Two-Component System; FHA, Fork Head-Associated domain; BTA, Bacterial Transcriptional Activation domain; T or Thr, Threonine residue; S or Ser, Serine residue; C, Cysteine residue. Asterisk (*) denotes the use of a heterologous *E. coli* over-expression system to determine *in vivo* phosphorylation. GBS, Group B Streptococci (*S. agalactiae*); GAS, Group A Streptococci (*S. pyogenes*).

b References where the phospho-modifications were identified.

### eSTK structure and function

**Figure 2.**
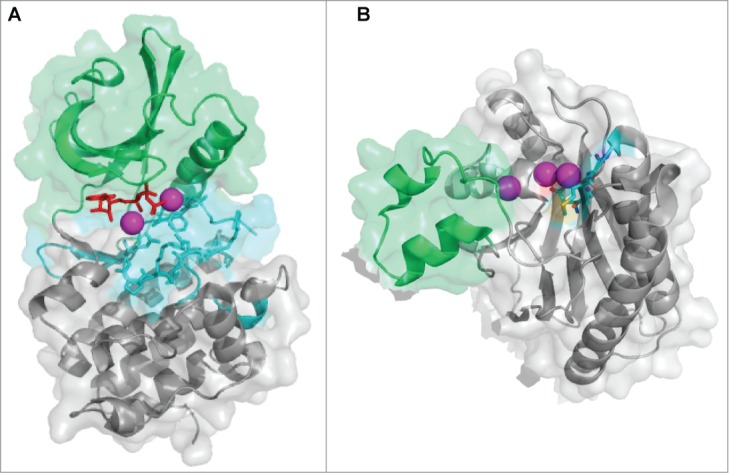
Atomic structures of eSTKs and eSTPs. (**A**) 3.0 Å atomic structure cartoon/surface of the PknB eSTK from *M. tuberculosis* (PDB code 1MRU; ref. 21). The two distinct lobes of the structure can easily be seen: (i) the N-terminal PKA subdomain consisting primarily of β-strands (colored green), and (ii) the a C-terminal subdomain almost entirely composed of α-helices (colored gray). Residues that make up the catalytic active pocket are colored in cyan. These residues include the metal (magnesium) binding domain, conserved Hank's-type ATPase active site motif “DXXPXN” and substrate interaction domain. The phosphothiophosphoric acid-adenylate ester (or AGS) substrate is colored red and magnesium ions colored magenta. (**B**) Cartoon/surface representation of the 2.65 Å atomic structure of the eSTP from *S. agalactiae* (PDB code 2PK0; ref.^48^). The Flap domain, thought to be involved with substrate interactions, is colored green. The conserved “DMGG” catalytic pocket motif that coordinates 2 of the 3 magnesium ions (colored magenta) is shown in cyan. In the DMGG motif the sulfur atoms are colored yellow, nitrogens blue and oxygens red.

The prokaryotic eSTK family was initially defined as cyclic AMP (cAMP)-dependent protein kinase (PKA) domains responsible for substrate recognition and phosphotransferase activity.^30^ Analysis of PknB in *M. tuberculosis*, the first structurally characterized bacterial eSTK PKA domain, reveals a typical bi-lobed organization strikingly similar to its eukaryotic versions^32-34^ (**Fig. 2A**). The structure possesses an N-terminal subdomain consisting primarily of β-strands followed by a C-terminal region almost entirely composed of α-helices, while the ATP binding pocket and substrate interaction site are sandwiched in between (**Fig. 2A**). This arrangement is also referred to as a Hanks-type domain VIb fold, which is defined by a conserved lysine present in the ATPase active site and the motif “DXXPXN,” where X is any amino acid.^30^ This architecture appears to be well conserved across the prokaryotic eSTK family, which suggests that a common mechanism of action is shared among these enzymes.^21^ However, extensive examination of the available bacterial genomes suggest that these receptor kinases can be further divided into several subclasses of related functions, and therefore might exhibit functional diversity similar to their eukaryotic counterparts.^31^

Although some eSTKs, especially those found in Gram-negative species, are predicted to be soluble cytoplasmic proteins,^35,36^ in Gram-positives most of these kinases possess a single transmembrane region that tethers the PKA module to a C-terminal extracellular sensory domain. This C-terminal sensory feature can take on many forms and is best exemplified by the unusually high number and diversity found in *M. tuberculosis*^22,35^^,37^ (11 total: PknA-B, and PknD-L). The multiple *M. tuberculosis* sensory domains include a β-propeller extension in PknD, a C-terminal Tpr and N-terminal rubredoxin-like sequence in PknG, a penicillin-binding protein and Ser/Thr kinase associated (PASTA) structure in PknB, and then finally several unidentified C-terminal domains in the remaining *M. tuberculosis* eSTK sequences (see **Fig. 6** and ref. 38 for a review of eSTK domain architecture in *M. tuberculosis*). Although historically the actual ligands that induce these diverse sensory domains have been limited to the PASTA repeats commonly found in other more canonical Gram-positives (see below), a number of recent studies have now shed further light on this subject. Convincing evidence has now shown that the beta-propeller extension in PknD is sensing osmolarity,^39^ which can change considerably, for example, during airborne transmission, when replicating within infected macrophages and within granulomas. In response to PknD osmotic stimulation, transcriptional machinery was activated in a kinase-dependent manner.^39^ Although the actual inducing ligand still remains at large, in the example of PknG experimentation has suggested that nitrated fatty acids (nitroalkenes) could be the natural interacting molecules.^167^ These compounds are metabolically produced during inflammation and could possibly serve as a means by which the bacteria can sense phagosome-lysosome fusion during macrophage intracellular replication.

Outside of the *Mycobacteria*, the most commonly studied eSTK extracellular domains have been PASTA repeats,^40^ which *Mycobacteria* also possess a homolog of (PknB) (**Fig. 1**). In contrast to the extensive conservation of the N-terminal catalytic core, PASTA domains show considerable disparity between homologs,^41^ suggesting that these repeats have evolved to accommodate variations in ligand structure. In support, some studies have indicated a surprising ability of PASTA domains to discriminate between different ligands, with apparently only slight changes in amino acid composition.^42^ However, given that the eSTK PASTA domains are homologous to known canonical penicillin-binding proteins (PBPs) whose function is the last catalytic step in peptidoglycan biosynthesis,^40^ it is unlikely that they have evolved for the primary purpose of detecting β-lactam-type antibiotics for which they are named. Nevertheless, PASTA domains are able to bind this class of antimicrobials with variable affinities.^43^

Thus far, the only known natural ligands of eSTKs described have been DAP-type hydrolysed muropeptide peptidoglycan fragments.^42,44^ As these findings have to date only been shown with the *Bacillus subtilis* PASTA-containing eSTK (PrkC),^42,44^ at the moment we can only infer that other homologs found within pathogenic bacteria have similar receptor specificity. Based on these seminal studies, the activation of eSTKs is thought to initiate by the binding of these muropeptide ligands, resulting in dimerization and subsequent autophosphorylation of the cytoplasmic N-terminal kinase domain. This action then results in phosphorylation of downstream target proteins, which eventually results in the modulation of transcriptional activity (**Fig. 1**; reviewed in ref.^29^). Intriguingly, recent work has demonstrated that PASTA-containing eSTKs might perform their functions in a specific area of the cell.^45^ Veening and colleagues have shown that in *S. pneumoniae* the PASTA-harboring eSTK (StkP) co-localizes to the bacterial divisosome with 2 of its known regulatory targets that control the cell division process, namely DivIVA and FtsA. Expanding on these studies, Grangeasse and colleagues have proposed that StkP, DivIVA and another protein called GpsB form a triad complex at the divisosome to enable the classic ellipsoid, or ‘cocci’ shape for which the pneumococcal cell is named.^168^ Collectively, these studies physically place PASTA-containing eSTKs at co-peripheral and septal cell-wall synthesis sites, and suggests that their function is, at least in part, to coordinate bacterial growth by modifying multiple substrates in this process with intricate timing.^45^

### eSTP structure and function

Differing from histidine kinases that often possess inherent phosphatase activity,^1^ eSTKs apparently lack this enzymatic function. This absent feature has presumably necessitated the evolution of an eSTP phosphatase counterpart to enable the option to revert the cell's physiology back to ‘normality’ following an eSTK phosphorylation-driven stress response. Interestingly, genomes with multiple eSTKs contain cognate eSTP phosphatases in far fewer numbers. For example, the *M. tuberculosis* genome possesses 11 eSTKs, yet only has a single predicted eSTP.^22^

The eSTPs belong to the protein phosphatase metal (PPM) binding family of enzymes, which are further subdivided into the PP2C class based on the presence of 11 conserved domains.^46^ Although classical PP2C phosphatases found in plants and other eukaryotes employ a bi-nuclear magnesium or manganese active site, structural analysis has revealed up to 3 metal ions bound within the catalytic core of the bacterial versions^47-49^ (**Fig. 2B**). The conserved eSTP active site is surrounded by a central β-sandwich and flanked by a pair of α-helices, which strikingly parallels the architecture of human versions^50,51^ (**Fig. 2B**). A protruding so-called flexible ‘flap’ domain is the major divergent structural feature from eukaryotic versions whose function is thought to involve substrate specificity and modulation of catalytic activity^49,52^ (shown in green in **Fig. 2B**). While their precise regulatory roles and consequences remain less understood than their kinase counterparts, available data show that eSTPs regulate the same vital eSTK processes in many pathogenic bacteria including cell division, virulence, antibiotic susceptibility and expression of virulence factors.^10-12,19,25,29,53^ An interesting recent report by Pancholi and colleagues has expanded the role of eSTPs by demonstrating that the *S. pyogenes* version is somehow secreted, and can then enter the host cell and nuclear envelope to affect apoptotic signaling cascades.^54^ Although important in their own right in the regulation of pathogen virulence traits, the scope of this review will focus more on eSTK regulation of transcription.

## Bacterial Transcription Factors

### Two-component systems

TCS systems are defined by their ability to accept a phosphate from a cognate histidine kinase partner on a conserved aspartate residue within the REC domain of the protein.^1,3,^^55^ Although REC domains can be associated with diversified output domains, they are most often found attached to helix-turn-helix (HTH) motif-containing effectors whose direct role is to bind DNA and modulate RNAP recruitment.^56^ REC domains can be found N-terminal to HTH effector domains as in the case of XRE, ArsR, HTH-10 and HTH-REC outputs. Alternatively, they can be found C-terminal to the effector module as in the case of TCS OmpR-type regulators having winged HTH DNA-binding motifs,^57^ and also BetR, AlpA, HlxR, LysR and MerR HTH-containing sequences.^56^

**Figure 3.**
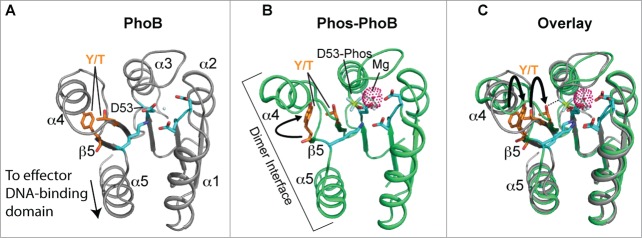
Structure and activation of REC domains. (**A**) Structure of the PhoB 2-component response regulator REC domain from *Escherichia coli* (PDB 1B00; ref. 158) shown in the inactivated, unphosphorylated state. PhoB exhibits a classical REC (α/β)5 topology, and α4-β5-α5 canonical dimeric interface. The proposed alternative dimer interface is the α1-α5.^63^^,169^ The tyrosine and threonine involved in ‘Y/T’ coupling are colored in orange. Notice the tyrosine is protruding into the solvent area of the α4-β5-α5 face to presumably prevent dimerization and activation. Key residues involved in the active pocket phosphorylation events are colored cyan, including the invariant aspartate residue (D53) that is phosphorylated by the cognate histidine kinase. Nitrogen atoms are colored blue and oxygen atoms red. (**B**) Structure of activated, phosphorylated PhoB (Phospho-PhoB; PDB 1ZES; ref. 62). Upon D53 phosphorylation, the conserved Y/T residues move toward the catalytic pocket (the Y movement is denoted by the arrow) to coordinate the new phosphate group (colored yellow) and allow the dimer to form. The magnesium ion and surrounding electron cloud (Mg) is shown in magenta. (**C**) Overlay of PhoB and Phospho-PhoB structures from *A* and *B*. The black arrows indicate the movements of the Y/T residues to allow dimerization about the α4-β5-α5 interface.

The three-dimensional structure of the canonical REC domain maintains an overall (α/β)5 topology (**Fig. 3A**; refs.^58,55^). These domains consist of 2 major areas of activity: (i) an acidic catalytic core containing the conserved Asp residue that receives the phosphate from the activated histidine kinase (*e.g.* Asp53 in PhoB, **Fig. 3A**), and (ii) a conserved Ser/Thr-Phe/Tyr pair which act as a molecular switch to enable dimerization and activation after the PTM has occurred.^55,^^59^ The latter events have been dubbed ‘Y/T-coupling’ and are responsible for driving the post-phosphorylation repositioning of the conserved Ser or Thr residue into the catalytic portion of the protein to help coordinate the newly-formed phospho-aspartate PTM (**Fig. 3B,C**). This repositioning then allows an invariable Tyr or Phe residue to also move ‘inwards’ to a less solvent-exposed area, which in turn leaves a more statically-favorable α4−β5−α5 dimeric interface for sister REC domains to interact and subsequently transmit the signal to the downstream effector domains (e.g., a HTH-containing motif) via an uncoupling of REC-effector hydrogen bonding.^60,61^ Once the REC-effector dissociation occurs, the REC C-terminal α5 helix linker is thought to become more flexible. This action then releases the DNA-binding motif to enable recruitment or obstruction of transcription through interactions with sigma (σ) factors and/or RNAP.^58^ Interestingly, an alternative dimerization site has now been identified within the α1−α5 helical face of the REC structure,^62,63^^,169^ and could be important for dimer-to-dimer contacts at promoters, providing additional and more complex options for bacteria to control their transcription.

Taken together, TCS response regulator modulation of transcription continues to show an increasing complexity and likely depends on the precise positioning of the transcription factors on the promoter, which can be influenced by variable environmental conditions.^64^ Previous genetic and biochemical data, combined with a low-resolution quaternary structure of the TCS response regulator PhoB bound to DNA, σ70 and the RNAP flap domain suggest that this complexity is controlled in part through direct interaction of the TCS regulator's transactivation loop located within the DNA-binding domain of the protein.^65,66^

### One-component systems

The so-called ‘One-Component’ Signaling (OCS) systems numerically dominate bacterial genomes and are defined by combining both input (sensory) and output (effector) modules into a single functional polypeptide.^51^ Like TCS effectors, these lone signal transduction proteins are usually transcription factors that contain an HTH motif (approximately 87%).^51,67^ However, OCS systems differ in that they exhibit greater diversity than their TCS counterparts. Although too numerous to list, OCS modules include more familiar and evolutionarily expansive protein families such as LysR, MarR and TetR-type regulators, as well as more understudied and rare HTH effector motifs. Not being constrained by upstream cognate partners, OCS systems are able to incorporate a wide range of inputs that include metal binding domains and small ligand effectors. For a comprehensive list of these bacterial signaling domains see references 51 and 67. Interestingly, some TCS system regulators have lost their conserved REC domain phospho-accepting aspartate residues and instead have evolved to accommodate more diverse inputs, effectively reverting back to a one-component unit.^55^ To date, examples of these pseudo-response regulators have been few, however they are important in that most described thus far are required for infection in a variety of pathogens, including RitR from *Streptococcus pneumoniae* that regulates iron uptake and related responses to reactive oxygen species (ROS),^69^ the general transcription regulator AmiR from *Pseudomonas aeruginosa* that regulates transcriptional termination,^68^ and HP1043 from *Helicobacter pylori* and ChxR from *Chlamydia trachomatis* that regulate infection and virulence traits.^70,71^

In this review we will concentrate on the emerging, alternative form of RNA synthesis control by eSTK phosphorylation of bacterial transcription factors, and the potential mechanistic consequences of these phospho-modifications. Emerging regulatory PTMs including the ability of eSTKs to phosphorylate tyrosines, as well as the new arginine kinases will not be covered in this review as they have recently been described in detail elsewhere.^72,73^ Here we will focus on eSTK phosphorylation transcription factors on serines, threonines and cysteines to enable timely control of important for virulence and infection traits in pathogenic bacteria (summarized in **Table 1**). In this regard, the future advancement of the field of prokaryotic PTMs will also be discussed, as a better understanding of these signaling systems will shed light on how eSTK/Ps empower bacteria to cause a range of infections in most important pathogens, and could ultimately offer a realistic focal point of therapeutic strategies in disease prevention.^74-77,146^

## Transcription Factors Regulated by eSTKs and eSTPs

### Staphylococcus aureus

**Figure 4.**
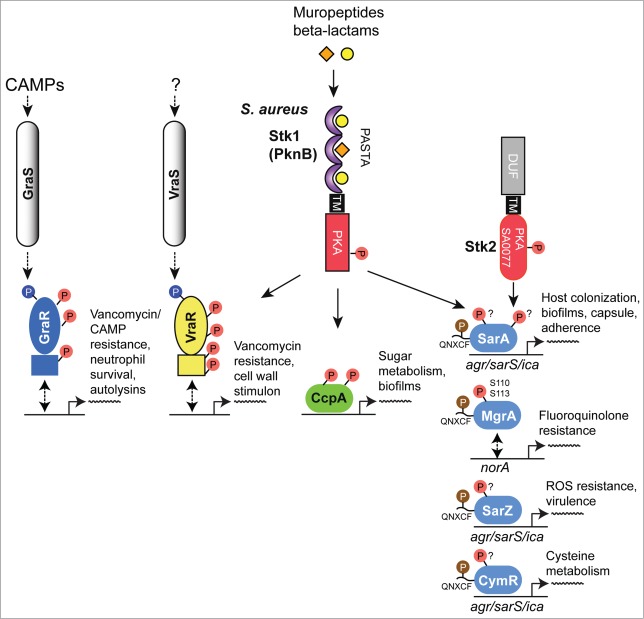
Overview of eSTK control of transcription in *S. aureus*. Schematic diagram showing the signaling pathways involved in eSTK control of *S. aureus* regulatory factors mentioned in this text. Phosphorylation is denoted by the circle with a “P.” Red circles indicate serine or threonine phosphorylation, brown cysteine phosphorylation and blue aspartate phosphorylation. Virulence and other traits that the regulators control are also described, as are regulated operons (if known). The TCS regulators GraR and VraR are phosphorylated by Stk1, as are the OCS regulators CcpA, MgrA and SarA. MgrA is phosphorylated by Stk1 on residues Ser110 and Ser113, and homologous sites yet to be verified experimentally are found in SarA and SarZ (see **Fig. 5**). However, aside from being phosphorylated by Stk1, SarA is also phosphorylated by the SA0077 (Stk2) kinase on threonine residues. Stk2 is only present in some *S aureus* strains harboring methicillin resistance. The question mark labels indicate that the exact site(s) of phosphorylation has not been identified. TM, transmembrane region.

As a human pathogen responsible for a wide range of infections, *S. aureus* is of major clinical significance.^78^ Underlying this clinical significance is this organism's virulence potential, as well as the increasing difficulties in providing treatment associated with antibiotic resistance. Indeed, the 2 eSTKs in this organism have been strongly implicated in both of these issues^79^ (reviewed in ref.^14^). To date, several *S. aureus* eSTK transcriptional targets have been identified that control this important pathogen's ability to survive, cause invasive disease and avoid the deleterious effects of antibiotic treatment. These are summarized in **Fig. 4**.

**Figure 5.**
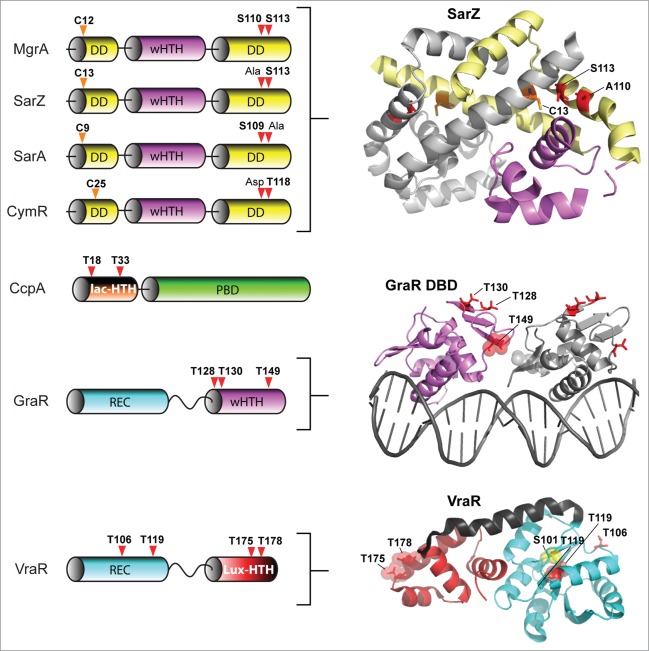
eSTK phosphorylation sites in *S. aureus* transcription. Domain architectures and identified phospho-residues (Serine (S), Threonine (T) or Cysteine (C), indicated by the arrows) are shown on the left. Some corresponding published atomic structures, or structural models that we have generated here as part of this review are shown on the right. Important residues are indicated in the structures. MgrA, SarZ, SarA and CymR all belong to the same family of transcription factors referred to here as the SarA/MgrA family and are regulated by phosphorylation of a conserved N-terminal cysteine residue (ref. 88). Only MgrA has been verified as having 2 additional eSTK-regulated sites (Ser110 and Ser113 within the C-terminal dimerization domain; ref. 82). Homologous Ser110/113 sites in SarZ, SarA and CymR regulators were found using 1- and 3-dimensional alignments. DD, dimerization domain; wHTH, winged helix-turn-helix domain; lac-HTH, LacI family HTH domain; Lux-HTH, LuxR family HTH domain; PBD, periplasmic binding domain; REC, receiver domain. Structures that are shown as dimers are depicted with one monomeric unit in color, and the other sister monomeric unit in gray. Phosphorylated residues are shown in red, except the phosphorylated cysteine in the SarZ structure is shown in orange, and the Ser101 and T119 sites in the VraR structure are colored yellow. The SarZ and VraR PDB codes used were 3HSE (ref. 159) and 4GVP (ref. 63), respectively. The GraR DNA-binding domain (DBD) atomic model was generated using a PhoB structure as a molecular template (PDB 1GXP; ref. 160) with Swiss-Model (ref. 161). All atomic figures were created with MacPyMol (ref. 162).

An early observation of eSTK/P-mediated modulation of transcription in *S. aureus* implicated the global transcription regulator MgrA, a member of the SarA/MgrA family of OCS regulators that contain a dimerization domain and winged HTH motif. However, little is known about how their regulatory effects are modulated in the absence of an obvious sensory input domain.^80^ What is known is MgrA is responsible for regulating several virulence factors that include α toxin, nuclease, lipase, coagulase and protein A, as well as autolysins and levels of capsular polysaccharide in *S. aureus*. In addition, MgrA also controls the synthesis of several efflux pumps involved in antimicrobial resistance in this pathogen.^81^ Work by Hooper and colleagues has shown that addition of *S. aureus* extracts to purified MgrA protein resulted in an abrogation of its binding to target DNA, which suggested PTMs were responsible. A later publication by the same group identified 2 adjacent serines (Ser110 and Ser113) as being the residues modified by the *S. aureus* PASTA-containing eSTK Stk1 (also referred to as PknB) and the cognate eSTP RsbU, which reciprocally regulates transcription of the genes encoding 2 drug efflux pumps (norA and norB), and consequently fluoroquinolone resistance^82^ (**Fig. 4**). Interestingly, Ser110 and Ser113 reside within the dimerization domain interface of MgrA (**Fig. 5**). This positioning is suggestive of a mechanism by which phosphorylation of these residues prevents MgrA dimerization and binding to DNA to allow derepression and therefore subsequent activation of the *norA* efflux pump under antibiotic imposed stresses.

Another *S. aureus* global transcription regulator SarA,^83^ a member of the SarA/MgrA family of prokaryotic transcription factors which modulates the expression of over 100 genes in this organism, has also revealed to be directly regulated via eSTK phosphorylation.^84^ Many of the genes that SarA controls include important virulence determinants such as *hla* (α toxin) and *spa* (Spa immune evasion molecule), the master virulence-controlling quorum sensing locus agr (accessory gene regulator), and sarS (staphylococcus accessory regulator), as well as biofilm formation through the regulation of the *ica* operon to facilitate host colonization.^85^ This important regulatory element also controls a plethora of other virulence determinants that include toxins (*e.g.* hemolysins and proteolytic exotoxins), synthesis of the capsule and adherence.^80,86^ Interestingly, a study by Didier et al. reveals that SarA is phosphorylated by *2* different *S. aureus* eSTKs. One, Stk1 (see above), controls SarA through threonine-specific phosphorylation, which greatly enhances its *in vitro* DNA-protein complex formation at promoters of key regulatory determinants *agr* and *sar*.^84^ Intriguingly, the second kinase that phosphorylates SarA, referred to by its gene annotation SA0077 and also called Stk2, only targets serine residue(s) in the protein (**Fig. 4**). Interestingly, SA0077/Stk2 is strain specific and only found thus far within a subset of methicillin-resistant *S. aureus* genomes (e.g., N315; ref. 166).

The domain architecture of SA0077 contains 502 amino acids, an N-terminal protein kinase domain, and similar to PASTA-containing versions like Stk1 possesses a single predicted transmembrane segment. However, C-terminal to the transmembrane helix is a predicted helical region of ∼200 amino acids with no homology to known domains or functionally characterized architectures. Contrary to the Stk1 regulatory effect on SarA, phosphorylation by the SA0077 kinase instead produces a decrease in DNA binding to known SarA regulated promoters.^84^ Although the precise sites of SarA phosphorylation by the SA0077 kinase were not identified, alignment of the available MgrA structure with that of the related SarZ (PDB IDs 2BV6 and 3HSE, respectively; structural alignment = RMS 2.09 Å, 117 to 117 atoms aligned; **Fig. 5** and data not shown) does show that the related SarA, SarZ and CymR regulators also possess a serine residue at one of the identified MgrA Stk1 phosphorylated positions (Ser110 or Ser113 equivalents; **Fig. 5**).^82^ Collectively these observations allude to a conservation of eSTK recognition in these related transcription factors. These findings are also particularly significant in that Didier et al. demonstrate the potential for multiple eSTK modulations of a single bacterial transcription factor, whose modifications affect the activation of different promoters. These studies add to the rapidly emerging picture of a more complex bacterial transcriptional regulatory network than previously anticipated. Further work is required to determine the precise effects of these phosphoserine PTMs with respect to infection and virulence.

A surprising additional complexity to this story was added by He and colleagues, where they found that the SarA and MgrA regulators in *S. aureus* act as *bona fide* functional redox sensors in this pathogen.^87,88^ Following this result, the same group made the seminal discovery that SarA, MgrA and related regulator SarZ are also controlled through a novel cysteine phosphorylation PTM via an apparently conserved “[Q/N]XCF” motif found within the N-terminus of these proteins^87,88^ (**Figs. 4 and 5**). The authors suspect that this novel PTM was missed in previous work due to standard conditions that are normally used in preparation of biological samples for mass spectrometry analysis (*e.g.* the use of reducing agents such as DTT). This thorough report (ref.^88^), which includes both *in vivo* and *in vitro* evidence as well as structural analysis, demonstrates that Stk1 is responsible for the observed cysteine phosphorylations, and ultimately enables the regulation of several virulence traits, as well as vancomycin resistance in response to oxygen and ROS. Strikingly, cysteine phosphoylation and associated regulatory effects can be readily reversed under reducing conditions. In this same report CymR, an additional SarA/MgrA family member from *S. aureus* that regulates cysteine metabolism in response to oxygen,^89^ was also found to be phospho-regulated by Stk1 through a similar N-terminal cysteine residue.^88^ A collective model for virulence factor regulatory control is thus proposed, whereby cysteine oxidation and phosphorylation act together to control dissociation of SarA/MgrA regulators from target promoters, which results in the activation of virulence factors (e.g., hemolysin^88^) (**Figs. 4 and 5**). An interesting area of future work will be to extend investigations of this novel PTM to other transcription factors and microbial species. We anticipate that the use of cysteine phosphorylation by pathogenic microbes to control virulence determinants in response to changing oxygen/ROS levels will open up an exciting new area of research in the field of bacterial signal transduction.

Another eSTK/P regulated pathway in *S. aureus* centers on the LacI/GalR family conserved transcription factor Catabolite control protein A (CcpA) that regulates sugar-related central metabolism in many bacterial species. In this pathway glycolytic intermediates are sensed by the HprK kinase, which, in turn, phosphorylates the intermediate Hpr relay protein on Ser46 to enable complex formation with CcpA and subsequent recognition of Catabolite-responsive elements (CREs) in target promoters.^90-92^ However, in addition to its well-established role in the regulation of metabolism, CcpA is also responsible for controlling virulence determinants (*e.g.* biofilm formation) in *S. aureus* and many other bacterial pathogens.^93-96^ In *S. aureus*, *in viv*o and *in vitro* studies have recently shown that phosphorylation of CcpA by Stk1 on 2 threonine residues (Thr18 and Thr33; **Figs. 4 and 5**) controls its function. These residues are located within the CcpA DNA-binding domain and are predicted to hydrogen bond with target DNA.^97^ In this example, eSTK phosphorylation of CcpA affords the bacteria a simple additional mechanism of regulatory control, whereby protein-DNA interactions can be directly abrogated by the PTMs to allow activation of CcpA-repressed catabolite promoters. Leiba et al. went on to show affected phenotypes through Thr18/33 regulatory control, including formation of biofilms *in vitro*.^97^ Interestingly, despite the exceptional conservation of Thr18/33 across multiple bacterial species, CcpA orthologs are unable to accept a phosphate by their native eSTKs, however cross-species phosphorylation is possible.^97^ Considering the high homology of eSTKs and CcpA orthologs across Gram-positive species, these surprising results suggest that slight changes within amino acid composition might afford a rapid reactivation of ‘dormant‘ eSTK controlled regulatory circuits. How such subtle discrimination by eSTKs between homologous variants of CcpA and other eSTK-regulated substrates is possible when all possess the conserved phosphorylatable residues remains an exciting topic of future research.

In addition to eSTK/P mediated reversible regulation of many OCS systems in *S. aureus*, several TCS systems are also regulated by phosphoserine-threonine PTMs in this pathogen. One such example comes from the response regulator GraR (for glycopeptide resistance associated), part of the GraSR TCS system associated with expression of the major *S. aureus* autolysin AtlA, survival within neutrophils, resistance to vancomycin and host cationic antimicrobial peptides (CAMPs), and virulence according to several *in vivo* infection models.^98-100^ The GraSR TCS is thought to be ligand-activated by host CAMP interaction with the histidine kinase GraS and the CAMP permease VraG to induce D-alanylation of teichoic acids and MprF-dependent lysylination of phosphatidyl-glycerol (**Fig. 4**). These modifications then collectively result in an increase in the bacterial surface positive charge and subsequent repulsion of CAMPs.^100^ On the other hand, besides the noted accumulation of mutations within the GraR response regulator that somehow enable incremental resistance to vancomycin, the precise mechanism of how GraSR conveys resistance to treatment with this antimicrobial still remains largely enigmatic.^101^ What is known is that GraR is another substrate of the *S. aureus* PASTA-containing eSTK Stk1.^102^

By employing *in vitro* kinase reactions combined with tandem mass spectrometry analyses, Fridman et al. show that GraR phosphorylation by Stk1 occurs on 3 threonine residues, namely Thr128, Thr130 and Thr149^102^ (**Fig. 5**). Importantly, experiments reveal that these residues are required for efficient incorporation of D-ala into the bacterial cell wall, which could directly affect the efficiency of vancomycin binding and explain the GraR connection with vancomycin resistance. Additionally, the phosphorylation of these residues enhances the binding of GraR to the *vraFG* CAMP ABC transporter promoter to control its synthesis^102^ (**Fig. 4**). A model of the GraR DNA-binding domain (shown as a dimer bound to DNA in **Fig. 5**) places residues Thr128 and Thr130 within the N-terminal antiparallel β-sheet, a structural element known to influence REC-DNA binding domain *intra*-protein contacts.^61^ Thus phosphorylation of these sites could potentially hold the 2 domains (REC and DNA-binding domain) apart in an extended conformation to instead allow a shifting of the structural equilibrium in favor of REC dimerization, and subsequent DNA-binding domain interaction with target DNA. Interestingly, Thr149 is predicted to face the other sister HTH motif, and when phosphorylated could thus influence these *inter*-protein interactions (**Fig. 5**).

To demonstrate eSTK specificity for the GraR regulator 2 additional Stk1 substrates were used: (1) the related OmpR-type TCS regulator BceR from *Bacillus subtilis* that confers bacitracin resistance and (2) the NarL family regulator VraR from *S. aureus* that confers vancomycin resistance and coordinates the cell wall stimulon. Consistent with the interspecies kinase assays described for eSTK phosphorylation of CcpA (above), despite the similarity of these regulators and the conservation of serine-threonine residues at the same positions found in BceR, both were unable to accept a phosphate from the *S. aureus* eSTK *in vitro*.^102^ Thus identification of conserved binding sites only indicates a *potential* for eSTK regulation, and therefore must be treated with caution. Future directions might include BceR and/or VraR variants from different strains to be tested, as sequences between clinical isolates can vary considerably and could therefore ‘unmask’ substrate specificity though subtle changes in amino acid composition.

In apparent contradiction to the aforementioned results, Molle and colleagues have demonstrated that VraR can indeed be phosphorylated by the *S. aureus* Stk1 *in vitro*.^103^ In these experiments mass spectrometry and mutagenesis were used to identify 4 phospho-accepting VraR residues: Thr106, Thr119, Thr175 and Thr178.^103^ Similar to parallel studies with CcpA, eSTK phosphorylation negatively impacts the DNA-binding properties of VraR. To explain these results, Thr175 and Thr178 are indeed positioned within the DNA-binding domain where they could inflict charge clashes between conserved protein-DNA or protein-protein contacts (**Fig. 5**). However, for the modified residues found within the REC domain, Thr106 and Thr119, the potential mechanistic contributions are less obvious. Indeed structures provided by Stock and colleagues are now available for both the inactive and active forms of the *S. aureus* VraR TCS regulator.^63^ An interesting feature is the position of Thr119, which is completely buried within the inactive (*i.e.* unphosphorylated aspartate) VraR structure (**Figs. 5 and 9B** below) and hydrogen bonds to the β5 strand (Ser101) located within the canonical α4−β5−α5 dimeric interface. Upon aspartate phosphorylation and the ensuing REC-DNA binding domain dissociation, Thr119 then becomes solvent-exposed.^63^ A likely *in vivo* mechanism one could envision is when VraR is activated by canonical Asp phosphorylation, the *S. aureus* Stk1 would then have ample access to phosphorylate the now solvent-accessible Thr119 and prevent unwanted reversion back to the inactive state. The *S. aureus* eSTP could then work to reverse this process under specific required circumstances (*i.e.,* removal of the PTM).

For the second phosphorylated threonine residue in the VraR REC domain (Thr106, **Fig. 5 and 9B**), in the *inactive* state there are no observed intra- or inter-protomer steric clashes, and its solvent exposure would therefore presumably afford easy accessibility to Stk1 interaction. However in the *active* (*i.e.* phosphorylated aspartate) state, this residue moves close to the other, alternative VraR α1−α5 dimerization face of the REC structure.^62,63^^,169^ A possible mechanism of activation inhibition was therefore suggested whereby Thr106 modification could interfere with formation of the VraR dimer at the α1−α5 interface by impairing crucial hydrophobic contacts.^103^

From these observations we can speculate that such simultaneous eSTK control of 2 different VraR dimer interfaces offers a glimpse of how eSTKs and eSTPs might further fine-tune canonical phospho-asparate TCS gene regulation to achieve a more specified phenotypic outcome. Finally, to add further complexity to this system, through elegant biochemical and genetic experiments by Msadek and colleagues it has been shown that in addition to VraR, the *S. aureus* GraR response regulator also directly interacts with the VraFG transporter promoter (**Fig. 4**), and the GraS histidine kinase directly binds the transporter itself.^100^ At present Canova et al. convincingly show that the previously mentioned eSTK-phosphorylated VraR residues are crucial for regulation of antibiotic resistance.^103^ It will be interesting to understand how these 2 interacting TCS signaling systems (*i.e.,* VraRS and GraRS), both of which are regulated by eSTK/Ps, work together to enable cationic peptide and vancomycin resistance in such an important clinically-relevant pathogen.

### Mycobacterium tuberculosis

**Figure 6.**
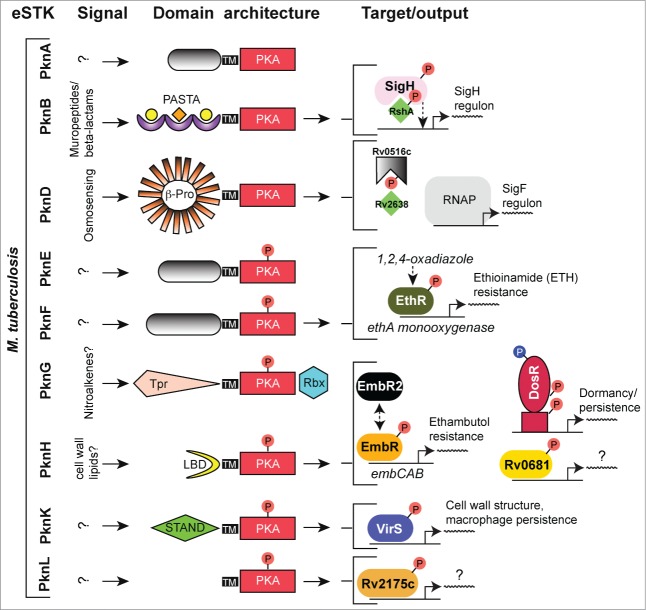
Overview of eSTK control of transcription in *M. tuberculosis*. Schematic diagram showing the signaling pathways involved in eSTK control of *M. tuberculosis* regulatory factors mentioned in this text. Phosphorylation is denoted by the circle with a “P.” Red circles indicate serine or threonine phosphorylation and blue aspartate phosphorylation. Virulence and other traits that the regulators control is described, as are regulated operons (if known). LBD, lipid-binding domain; β-Pro, β-propeller domain; TM, transmembrane region. Other abbreviations are also described in the text.

*M. tuberculosis* is the causative agent of one of the oldest recorded afflictions of mankind, and is responsible for an annual death toll of millions worldwide. The increasing difficulties associated with its treatment have, in one respect, been attributed to the bacteria's ability to enter a state of dormancy and thus persist within the host. This issue coupled with an alarming increase in antibiotic resistance has now enabled this pathogen to become a source of high morbidity and mortality worldwide.^104^ A large part of this pathogen's ability to regulate antibiotic resistance and its persistent lifestyle in the host can be attributed to the 11 eSTKs (named PknA through PknL), the single eSTP (called MstP), and a growing list of important eSTK/P regulatory targets (reviewed in refs.^6,22^). Within this list of regulated proteins are several transcription factors that play crucial roles in the pathogenicity and virulence of *M. tuberculosis* infection (summarized in **Fig. 6**).

**Figure 7.**
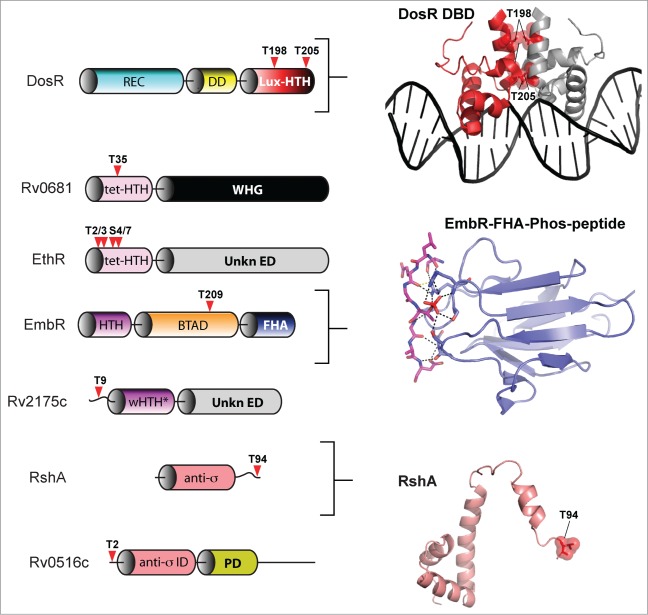
eSTK phosphorylation sites in *M. tuberculosis* transcription factors. Annotation is as per **Figure 4**. Domain architectures are shown on the left, and 3-dimensional atomic structures and models on the right. The PDB codes for the DosR DNA-binding domain (DBD) and the EmbR-forkhead-associated domain (FHA; shown in blue) in complex with an interacting peptide (in magenta) and phosphate (shown in red) are 3C3W (ref. 163) and 2FF4 (ref. 114), respectively. The RshA anti σ-factor structure was generated with Swiss-Model using an available crystal structure from the anti-sigma factor ChrR (PDB code 2Q1Z; ref. 164) as a molecular template. eSTK phosphorylated residues are shown in red. Abbreviations not previous described are tet-HTH, TetR family helix-turn-helix domain; WHG, tryptophan-histidine-glycine domain presumed to bind a ligand or unknown effector molecule; UnKn ED, unknown effector domain; BTAD, bacterial transcriptional activator domain; wHTH*, wHTH domain missing helix 3; anti-σ: anti-sigma factor; anti-σ ID, anti-sigma factor interacting domain; PD, phosphorylation domain.

The primary point of dormancy regulatory control in *M. tuberculosis* is mediated by the TCS-like response regulator DosR (also known as DevR and Rv3133c).^105^ The DosR regulon consists of over 50 genes that are activated in response to hypoxia, nitric oxide and carbon monoxide. These signals are collectively sensed by the 2 cognate histidine kinases DosS and DosT, which through aspartate phosphorylation of DosR control entry of *M. tuberculosis* into its persistent state.^106,107^ The discovery that DosR was also regulated by phosphorylation by the eSTK PknH on 2 threonine residues (Thr198 and Thr205) located within its C-terminal helical dimerization interface added an another regulatory complexity to this TCS-like system^108^ (**Figs. 6 and 7**). Phosphorylation of the DosR threonines has the effect of enhancing DosR DNA-binding affinity to a level comparable to a similar enhancement through Asp phosphorylation by the cognate histidine kinase DosS. Interestingly, DosR binding to its target DNA is synergistically enhanced by the simultaneous phosphorylation of its threonine residues and the canonical TCS aspartate, as compared to either PTM alone. These data collectively suggest that modulation of the *M. tuberculosis* hypoxia response is mediated by a convergence of eSTK and TCS regulated pathways, and that phosphorylation by both is required to achieve full activation of the DosR regulon.^108^ In general, the PknH kinase itself appears to respond to variations of environmental stresses. Treatment of *Mycobacteria* cells with nitric oxide leads to a two-fold induction of expression the DosR regulon under the control of PknH while exposure to acidic pH and excess heat also reduces PknH expression.^108^ However, a more recent report has come a step closer to finding the PknH ligand. Av-Gay and colleagues have found that disruption of PknH affects the synthesis of phthiocerol dimycocerosates (PDIMs), a major class of cell wall associated lipids in *M. tuberculosis* that are required for its virulence.^109^ Although the actual ligand for PknH remains at large, a crystal structure of the extracellular domain has revealed a large hydrophobic and polar cleft where cell wall related lipids could bind.^110^ Future work will have to determine the precise inducer of PknH activation and the subsequent downstream effects on regulation of the crucial TCS-like transcription factor DosR.

Of equal importance to mycobacterial persistence is its ability to resist antimicrobial treatments. The transcription factor EmbR is integral to these regulatory functions through the control of 3 genes, *embA-C*, which mediate resistance to ethambutol, a key frontline antitubercular drug that targets arabinosyltransferases.^111^ The products of the *embCAB* operon are involved in the arabinosylation of the cell wall component lipomannan to produce lipoarabinomannan, the former of which induces a potent proinflammatory response and are critical to the structure of the mycobacterial cell envelope.^111^ In the context of this review, the EmbR regulator is also historically important in that it was the first eSTK substrate to be identified in *M. tuberculosis*. EmbR is member of the *Streptomyces* antibiotic regulatory protein (SARP) family of transcription factors^112,113^ and maintains a unique structure consisting of an N-terminal OmpR-type DNA-binding domain, followed by a bacterial transcriptional activation (BTA) domain whose fold resembles that of the tetratricopeptid repeat (TPR) architecture of human phosphatases.^114^ An interesting feature of EmbR is its C-terminal forkhead-associated (FHA) domain, a conserved class of short protein motifs known to facilitate recognition of phospho-threonine-containing peptides, including those belonging to eSTKs^112^ (**Fig. 7**). Indeed, several key papers have now shown in great molecular detail how FHA domains can act as intramolecular inhibitors of target protein binding by association with a phospho-threonine group on a different portion of the FHA-containing protein.^114-116^ To help illustrate this interaction, **Fig. 7** shows an atomic structure of the EmbR FHA domain (ref. 114) bound to a phospho-threonine peptide.

In *M. tuberculosis* EmbR is also targeted by the PknH kinase, where it is phosphorylated on threonine residues to stimulate interaction with the *embCAB* promoter^111,112^ (**Fig. 6**). These PTMs can then be nullified upon truncation of the FHA domain, or by mutation of highly conserved residues within,^112^ highlighting its requirement for the EmbR-PknH interaction. Positive regulation of the *embCAB* operon through EmbR serine-threonine phosphorylation also occurs *in vivo*, which correlates with an increase in lipoarabinomannan levels and resistance to ethambutol. In an interesting twist to this story, a protein dubbed “EmbR2,” which is 56% identical to EmbR, competes with PknH as a substrate, yet is unable to accept a phosphate. Thus EmbR2 might act as a natural dominant negative regulator of the “real” EmbR to help facilitate an effective inhibition of PknH autophosphorylation.^117^ In addition to PknH, PknA and PknB are also capable of phosphorylating EmbR *in vitro*, which can be subsequently dephosphorylated by the cognate eSTP MstP.^118^ In these studies MstP driven dephosphorylation of EmbR dramatically reduces its DNA binding ability *in vitro*.^118^ Although these interactions and their relevance to *in vivo* regulation of EmbR have yet to be determined, like the SarA factor from *S. aureus* described above it is interesting to note that several eSTKs might be co-regulating a single transcription factor. Studies showing the precise modified amino acids, their mechanistic and phenotypic outcomes will be of interest in future studies.

*In silico* predictions have also proven useful for identification of potential phospho-targets of eSTKs. For example this methodology allowed Av-Gay and colleagues to identify targets of PknH, where it was reasoned that the kinase's substrate recognition motif, determined to be “TQLGT,” resembles the core autophosphorylation site of the eSTK itself.^119^ Forty PknH targets have been identified using this method, including the already characterized EmbR that served as a control, and the putative TetR family transcription factor dubbed “Rv0681.” In EmbR, mutating the predicted phosphorylated BTA domain residue Thr209 produces only a slight decrease in PknH phosphorylation *in vitro*, suggesting that additional Ser or Thr residues might be modified. Conversely, replacing the single predicted phospho-modified site within Rv0681, Thr35 within the DNA-binding domain, serves to completely abrogate PknH phosphotransfer to this substrate. These findings require verification as relevant *in vivo*. However, as the recognition sequences for different eSTK homologs are likely to vary,^26^ *in silico* and extensive proteomic experiments have thus far proven to be a powerful means from which to identify their pleiotropic substrates.^119^ In support, a comprehensive study using proteomics to identify eSTK targets and their associated phosphopeptides was able to generate a potential list of eSTK specific recognition motifs. These motifs could then presumably be used to conduct searches for additional eSTK substrates and thus provides a template for many studies to come.^26^

Another drug that is typically used to treat active tuberculosis is ethionamide (ETH). To be effective, ETH must be activated and its potency is enhanced through chemical modification by the monooxygenase EthA.^120^ Expression of *ethA* is, in turn, repressed by the TetR family transcription factor EthR, and thus by association EthR controls levels of ETH resistance in *Mycobacteria*^121^ (**Fig. 6**). In a recent publication, Molle and colleagues show that EthR is an *in vitro* substrate for the *M. tuberculosis* eSTK PknF.^122^ Mass spectrometry identifies 4 phosphorylated residues within the extreme and seemingly unstructured N-terminus of the protein (Thr2, Thr3, Ser4, and Ser7; **Fig. 7**). Phosphotransfer by PknF is only absent when either all 4 residues are substituted for, or alternatively can be accomplished by deleting the entire N-terminal sequence.^122^ Further work demonstrates that *in vitro* DNA-binding to the EthR target *ethA* promoter decreases in these serine-threonine mutant EthR versions compared to wild-type.^122^ Interestingly, the authors go on to show that in the presence of the potent EthR-targeting synthetic anti-mycobacterial antibiotic 1,2,4-oxadiazole, the observed negative effect PknF phosphorylation has on EthR DNA affinity is further enhanced by addition of the drug. From these data the authors proposed a 2-pronged mechanism of PknF mediation of EthR control. First the PTMs inhibit EthR from binding DNA through direct interference with its protein-DNA static interactions. Second, eSTK phosphorylation enhances the effect of 1,2,4-oxadiazole by increasing affinity for its allosteric binding site, which is located in a structurally distinct position from the EthR protein-DNA interface. Collectively, these data indicate a possible *M. tuberculosis* resistance mechanism for the 1,2,4-oxadiazole class of drugs mediated through eSTK transcription factor phospho-modification. Future studies will include using *in vivo* techniques and clinical isolates to further test the efficacy of these antibacterial compounds.^122^

Another *M. tuberculosis* eSTK targeted transcription factor, Rv2175c, thought to be involved in the control of important cell envelope related functions, was discovered through an initial proteomic-based study in search of novel kinase substrates.^123^ A follow-up study identifies Thr9 as being the sole residue in Rv2175c (**Fig. 7**), which is specifically phosphorylated by PknL. Although the exact inducing signal for PknL has yet to be determined, evidence suggests a role similar to that of PknA and PknB in cell wall associated functions, as well as the regulation of glutamine metabolism.^124^ An available structure of the PknL substrate Rv2175c reveals a surprising OmpR-like TCS winged helix-turn-helix (wHTH) DNA binding motif that is missing its typical third helix, a structural phenomenon usually only observed in phage-derived transcription factors. The wHTH is preceded by a flexible and disordered 16-residue long N-terminus that contains Thr9.^125^ DNA-binding activity can be completely abrogated by a Thr9 to Asp phospho-mimetic mutation, and yet remains intact after deletion of the first 13 N-terminal residues of the protein containing this residue. With the tertiary structure of the monomer remaining virtually unaffected by phosphorylation,^125^ it can be concluded that the PTM might affect dimerization, complex-forming interactions with RNAP or other cofactors at the bacterial promoter. Interestingly, the N-terminal regulated extension is unique to *M. tuberculosis*, not even found in other closely related mycobacterial species,^125^ which suggests species-specific eSTK control of the Rv2175c regulon. An interesting aspect of these results is that where a plethora of OCS transcription factors similar to Rv2175c have now been identified, it has remained enigmatic as to how they are regulated in the absence of an obvious input domain. This work by Cohen-Gonsaud, Molle and colleagues suggests the exciting prospect that such eSTK control over mycobacterial ‘DNA-binding domain only’ transcription factors might now offer a ubiquitous mechanism for their regulation.

Also contributing to the survival of host-inflicted stresses on *M. tuberculosis* is the AraC family transcription factor VirS. VirS is homologous to several virulence-regulating transcription factors found in other pathogens (e.g., *Shigella*) and related mycobacterial species, where it mediates the correct formation of the cell wall structure and protects against low pH conditions.^126^ The involvement of VirS in these stress responses is regulated through the mycobacterial eSTK PknK, an eSTK required for survival in the early stages of mycobacterial infection and persistence, and likely facilitates adaptation to intracellular environments through translational control^127^ (**Fig. 6**). Along with PknG, PknK is evolutionarily distinct from that of the other *M. tuberculosis* eSTKs in that it is widely distributed in prokaryotes, both pathogenic and non-pathogenic, and is a soluble protein. At present the signals that PknK responds to are not known, however the presence of a C-terminal STAND domain suggests it might be a signaling hub for many sensory inputs that remain to be elucidated.^127^ On the other hand some PknK substrates have been revealed. Using VirS protein produced in a heterologous *E. coli* expression system,^128^ Kuman et al. were able to show that PknK phosphorylates VirS. The phosphorylated protein exhibited a 2.5 fold higher affinity for the VirS target promoter of *mymA* that encodes a putative monooxygenase.^129^ Although the site(s) of phosphorylation have not been elucidated, PknK control of VirS was shown to be relevant *in vivo* by enabling a regulatory effect over *mymA* operon transcription.^129^ The same work by Singh et al. highlights the importance of *mymA* in controlling the mycobacterial cell wall ultrastructure, persistence within activated macrophages and the specific requirement for infection of the spleen but not the lungs (**Fig. 6**). These studies further highlight the individual roles mycobacterial eSTKs play in virulence and infection of diverse host tissues.

Although little data is available on the subject, some proteomic studies have implicated eSTKs in the regulation of transcription through direct phosphorylation of core transcriptional machinery. For example, by employing proteomic approaches in the human pathogen *S. pneumoniae*, the α subunit of RNAP was identified as a possible substrate of the native eSTK (StkP) in this organism.^23^ Similar techniques have also been used in *Listeria monocytogenes* to demonstrate interaction between a *Listeria* eSTK and the α and β subunits of the RNAP holoenzyme.^27^ Taken together, these findings suggest that eSTKs may use modification of transcription machinery components to influence gene expression on a global scale. However intriguing, data from these experiments have not been verified by additional biochemical and genetic methods. On the other hand, direct regulation of sigma factors by eSTKs and eSTPs and their mechanistic consequences have been demonstrated, especially within *M. tuberculosis*. One example includes SigH, the alternative sigma factor responsible for regulation of mycobacterial stress responses and required to achieve the full virulence potential of this organism according to a murine model of infection.^130^

The activity of SigH is post-translationally regulated via interaction with the anti-sigma factor RshA in an effort to control mycobacterial internal redox conditions.^130^ The SigH-RshA interaction occurs in a redox-dependent manner through 4 conserved cysteine residues within RshA. RshA is itself a substrate of the eSTK PknB and its phosphorylation interferes with the SigH interaction. This PTM might therefore serve to limit the activation of SigH dependent regulons.^131^ Combing extensive *in vivo* and *in vitro* experiments with mass spectrometry analysis, it was determined that RshA can be phosphorylated on just one threonine residue that is relevant to its *in vivo* activity (Thr94). Intriguingly, Thr94 is located near the RshA C-terminus by helix 4 and is a critical region for sigma factor/anti-sigma factor interactions (**Fig. 7**). eSTK-mediated phosphorylation of SigH can also be observed on 2 of its threonines (Thr26 and Thr102).^131^ However, differing from RshA phosphorylation, the SigH PTMs does not produce a pronounced disruption in SigH-RshA complex formation. Possible explanations for this discrepancy could be low phosphorylation stoichiometry, or that SigH phosphorylation may instead be specific for an as-of-yet unidentified non-RshA interacting partner.

*M. tuberculosis* also possesses many anti-anti-sigma factors that act to sequester and inhibit anti-sigma factors and promote RNAP recruitment under specific environmental circumstances. One of these, the predicted anti-anti-sigma factor Rv0516c, has been identified as a substrate of the eSTK PknD by Alber and colleagues^132^ (**Fig. 6**). The PTM occurs *in vitro* within the N-terminal sequence on Thr2, and also *in vivo* when over-expressed with the PknD kinase. Phosphorylation of Rv0516c results in an effective abolition of its interaction with its anti-anti-sigma factor Rv2638, and a subsequent activation of the SigF regulon that responds to osmotic shock^39^ (**Fig. 6**). In the same publication by Greenstein et al., the authors expand on these studies by testing a combination of 8 different putative mycobacterium anti-anti-sigma factor substrates for their ability to receive a phosphate from PknA, PknB, PknD, PknE, and PknK eSTKs.^132^ Results reveal a surprising specificity for these substrates by specific eSTKs. For example, PknA phosphorylates no anti-anti-sigma factors, whereas PnkB and PknE only phosphorylates a single substrate out of the 8 tested. This study shows the potential extensiveness of eSTK regulation of transcription in a single organism, and gives us a glimpse of how eSTKs might similarly control many signaling networks in most bacterial species with surprising discrimination.

### Streptococci

**Figure 8.**
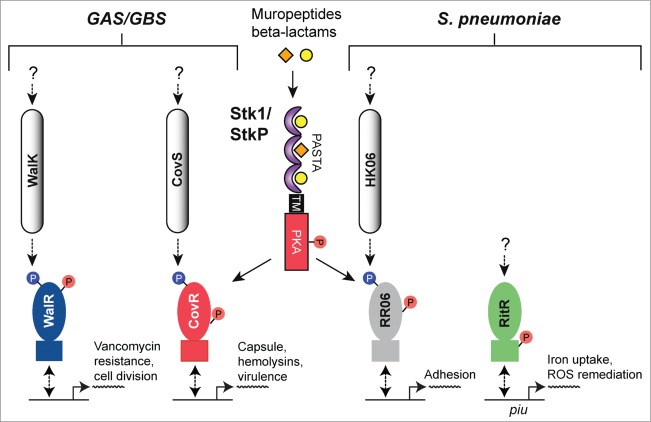
Overview of eSTK control of transcription in the streptococci. Schematic diagram showing the signaling pathways involved in eSTK control of Group A Streptococci (GAS), Group B Streptococci (GBS) and *S. pneumoniae* regulatory factors mentioned in this text. In GAS and GBS the PASTA-containing eSTK is called Stk1, and in *S. pneumoniae* StkP. Stk1 from both GAS and GBS modulates the TCS regulator CovR in these species, whereas WalR has thus far only been shown to be phosphorylated by Stk1 in GAS. Similarly, StkP modulates the TCS regulators RR06 and RitR in *S. pneumoniae*. The question mark shown above RitR indicates that aside form StkP phosphorylation, regulation from other input signals has not been identified. The phosphorylation PTM is denoted by the circle with a “P.” Red circles indicate serine or threonine phosphorylation, whereas blue indicates aspartate phosphorylation. Virulence and other traits that the regulators control are also described. Transmembrane region (TM). The TCS histidine kinases CovS, WalK and HK06 have 2 TMs each that are not annotated here for purposes of simplicity.

The *Streptococcus* encompasses numerous species of pathogenic Gram-positive bacteria, including but not limited to *S. pyogenes* (also referred to as Group A streptococci or GAS), *S. agalactiae* (also called Group B streptococci or GBS) and *S. pneumoniae*, all of which are capable of causing a diverse array of debilitating diseases. GAS, GBS and *S. pneumoniae* each possess only a single predicted PASTA domain-containing eSTK/eSTP pair, called Stk1 in GAS/GBS and StkP in *S. pneumoniae*, all of which are required for infection^7,8,13,25^ (reviewed by Burnside and Rajagopal in ref.^133^). The regulatory effects of eSTK phosphorylation in the streptococci are summarized in **Fig. 8**.

The CovR transcription factor (also referred to as CsrR) is part of the control of virulence (CovRS) TCS system that regulates a large number of genes in both GBS and GAS, including those involved with production of the capsule, major virulence factors, penetration of blood-tissue barriers and avoidance of the immune system^134-136^ (**Fig. 8**). For GBS, strains lacking their eSTK Stk1 were deficient in their ability to survive neutrophil attack and oxidative stress, and the ability to produce their key virulence factor β-hemolysin/cytotolysin (β-H/C).^137^ This landmark paper by Rajagopal et al. demonstrated for the first time that these processes are regulated by the convergence of the CovRS TCS system and an eSTK, and importantly is the initial example of such co-regulatory control in a pathogen. Subsequent work from the same group has demonstrated that the regulation of β-H/C is mediated through eSTK phosphorylation of Thr65 in CovR, which abolishes binding to the β-H/C promoter and in doing so, alleviates its inhibition on the expression of β-H/C.^138^ In contrast, phosphorylation of the conserved CovR aspartate residue (Asp53) by the cognate CovS histidine kinase serves to repress β-H/C production. A more recent study has further expanded on these findings to demonstrate how the homologous eSTK and CovRS TCS regulatory pathways in GAS converge to fine-tune global virulence traits through the same site (ref.^139^ and see below).

**Figure 9.**
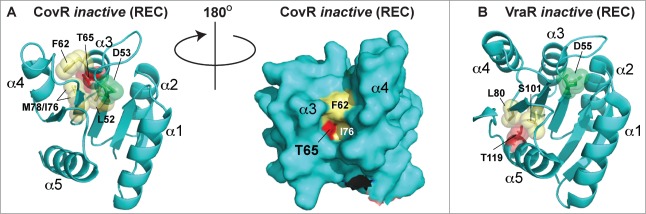
Comparison of CovR and VraR Receiver (REC) domain phosphorylation sites. (**A**) (left panel) Cartoon representation of an atomic model of the CovR REC domain. The model was created with Swiss-Model (ref.^161^) using the CovR sequence from *S. agalactiae* and the PhoP structure from *M. tuberculosis* (PDB code 3R0J; ref.^165^) as a molecular template. Phosphorylated residues are shown in red. The conserved histidine kinase phosphorylated aspartate (Asp53) is shown in green. Residues that would potentially pose a steric clash with the phosphorylated Thr65 are shown in yellow. (right panel) Surface representation of the left panel rotated 180 degrees. Notice that Thr65 is solvent accessible, but resides in a deep cleft within the interior of the protein. (**B**) The VraR atomic (REC) structure taken from ref.^63^ showing the eSTK-regulated Thr119 (colored red) and potential residues that might clash with it in its phosphorylated form (colored yellow). The conserved Asp55 is shown in green.

The eSTK-regulated Thr65 residue is located within helix 3 of CovR REC domain. Although an atomic structure of the CovR REC domain is not available, the model shown in **Fig. 9A** predicts Thr65 to reside within a deep cleft, facing the interior of the protein where the phospho-accepting oxygen is hydrogen-bonding to nearby residues. Although the eSTK substrate recognition domain is known to be flexible,^21^ Thr65 would seem difficult to access within this model static structure. For comparison, in the REC domain of the *S. aureus* regulator VraR the eSTK-modified Thr119 is completely solvent-inaccessible and facing the interior of the protein (**Fig. 9B** and data not shown). How eSTKs are able to access such residues is of great interest. One plausible explanation is REC domains exist in a dynamic equilibrium of “on/off” states, and similar to the canonical phosphorylation of the REC aspartate, a serine or threonine modification could shift this equilibrium toward the CovR protein “on” state rather than the “off."^140^ The predicted Thr65 position in 3-dimensional space is also interesting, where its electron cloud kisses that of the opposing Leu52 residue, the latter of which is located just N-terminal to the CovS-regulated Asp53 (**Fig. 9**). With this observation in mind we can envision the phosphorylation of Thr65 perturbing the interior of the REC domain, shifting the protein's equilibrium state and altering the position of the canonical phospho-Asp site in such a way as to disfavor CovS interaction and/or Asp53 modification.

Paralleling the aforementioned findings by Rajagopal and colleagues, Argwal et al. show that the eSTK/eSTP pair from *S. pyogenes* is also able to reversibly phosphorylate the endogenous CovR GAS homolog. Moreover, in the same work they identify the essential TCS regulator WalR, responsible for controlling crucial pathways involving cell wall metabolism and *in vivo* accumulation of multidrug resistance,^141^ as an additional eSTK/P target^10^ (for a review on the WalRK TCS see ref. 142). Both CovR and WalR *S. pyogenes* homologs are phosphorylated on threonine residues as determined by chromatography experiments (**Fig. 8**). Whereas the WalR modified site(s) have yet to be deciphered, the precise site of eSTK regulation of the GAS CovR is now known to be identical to that of the GBS homolog (*i.e.* Thr65). These recent and elegant studies by Horstmann et al. (ref. 139) not only demonstrate the relevance of Thr65 to GAS infection and virulence, but also importantly show the potential for eSTK/P cross-species conservation of regulatory control.

A very novel observation stemming from the studies by Pancholi and colleagues was that in infected pharyngeal cells the *S. pyogenes* eSTP was exported from the pathogen into the host nucleus to initiate pro-apoptotic events and a redirection of the host transcriptome.^54^ In further support of these findings, Miller et al. have previously identified the *S. aureus* eSTK PknB as a secreted protein, which was also shown to phosphorylate pro-apoptotic host proteins *in vitro*.^143^ eSTK and eSTP modulation of host proteins has also been described in *Yersinia* sp. *Salmonella* sp. and several other important bacterial pathogens.^144,145^ An area of research to expand upon would include determining how eSTK/Ps, especially those containing transmembrane regions and from species that do not possess typical effector secretion systems (*e.g.* type III), are able export eSTK/Ps out of the bacterial cell, into the host cytoplasm, and in some cases into the nucleus to enable direct rewiring of host transcriptional circuitry.

Despite an available vaccine, *S. pneumoniae* continues to be one of the leading causes of morbidity and mortality worldwide, especially in young children.^146^ Understanding how this organism is able to infect such a variety of tissues and cause disease is a crucial step in the progress of producing novel antibiotics and antigens for vaccines. A major focus of preventing pneumococcal disease is deciphering how it adheres to and colonizes the seemingly harsh environment of the nasopharynx, where it is exposed to unwanted oxygen and has to contend with the host immune system.^146,147^ In another study carried out by Pancholi and colleagues, the pneumococcal TCS response regulator RR06, which controls expression of adhesion elements,^148^ exhibits reversible regulation by the eSTK/P pair in this pathogen.^149^ Furthermore, phosphorylation of RR06 on threonine residue(s) results in added stability to its DNA binding (**Fig. 8**). However the site of modification and *in vivo* relevance of these modifications still remains at large. An interesting outcome from these studies was that the pneumococcal eSTK/P pair demonstrates control over its virulence properties in a strain-specific manner. These findings hint at how *S. pneumoniae* and other pathogenic bacteria might use eSTK/P regulation to alter required target specificity through evolving single amino acid changes in their substrates in response to changing host environments. In support, it has recently been reported that point mutations within the GAS homolog of CovR in certain clinical isolates have dramatic effects on expression of CovR-regulated virulence factors, pathogenesis and presumed clinical outcomes.^150^

During infection streptococci and all other pathogens are reliant on the host to provide their nutritional requirements or perish. For most bacterial pathogens their success is measured in part by host iron availability. Ironically, although iron is the fourth most abundant metal on earth, within the host environment it is surprisingly scarce, where it is found at concentrations far less than required to sustain bacterial growth (<10−9 M on average).^151^ However when obtained, iron must be kept distant from H_2_O_2_ to avoid killing by iron-catalyzed Fenton chemistry.^152^ In *S. pneumoniae* this balance is especially important, as it produces up to millimolar quantities of H_2_O_2_ as a metabolic byproduct of pyruvate oxidase activity in the presence of oxygen.^153^

Repressor of iron transport regulator (RitR) is a TCS-like response regulator in *S. pneumoniae* required for lung infection and responsible for iron uptake and remediation of iron-catalyzed ROS.^69,154,155^ These actions are carried out in part by RitR-mediated repression of the pneumococcal iron uptake (Piu) transporter, and its simultaneous activation of bacterial ferritin (Dpr) and DNA repair enzymes under high oxygen tension (ref.^69^ and unpublished data; **Fig. 8**). RitR is unusual in that it is a so-called orphan response regulator, which by definition does not possess a cognate histidine kinase partner. Instead RitR is co-transcribed with 6-phosphogluconate dehydrogenase, a key enzyme in the pentose phosphate carbon utilization pathway.^69^ Moreover, RitR is missing the conserved phosphorylated Asp residue within its REC domain. This posed the interesting question of how this transcription factor is regulated. To answer this question a randomized phage display library was employed to identify a peptide which specifically bound the RitR DNA-binding domain, and whose sequence was identical to the conserved catalytic pocket of the ubiquitous PP2C phosphatase family of proteins that includes eSTPs.^24^ Titration of purified pneumococcal eSTP to RitR bound to target DNA revealed the formation of a tripartite eSTP-RitR-DNA complex, which can be disrupted by addition of the cognate eSTK partner StkP.^24^ The pneumococcal eSTK (StkP) and eSTP (PhpP) were then shown to reversibly phosphorylate RitR within its DNA-binding domain to enable gene regulation of the Piu iron-heme transporter.^24^ Although the exact site(s) of RitR phospho-regulation remain unknown, these studies hint at how bacterial eSTK and eSTPs might be found in complex formation at the site of transcription initiation to direct its control.

Interestingly, several subsequent publications have linked eSTK and eSTP regulation with controlling intracellular iron concentrations and resistance to ROS, including resistance to macrophage and neutrophil oxidative killing in many pathogens (e.g., refs.^156,157,25^). As eSTK and eSTP regulated functions in the streptococci seem predominantly related to control of virulence factor release and cell envelope related functions, it currently remains unclear how they might also affect responses to a changing redox state, ROS and fluctuating metal ion concentrations. This remains an exciting topic for future investigations.

## Future Perspectives

**Figure 10.**
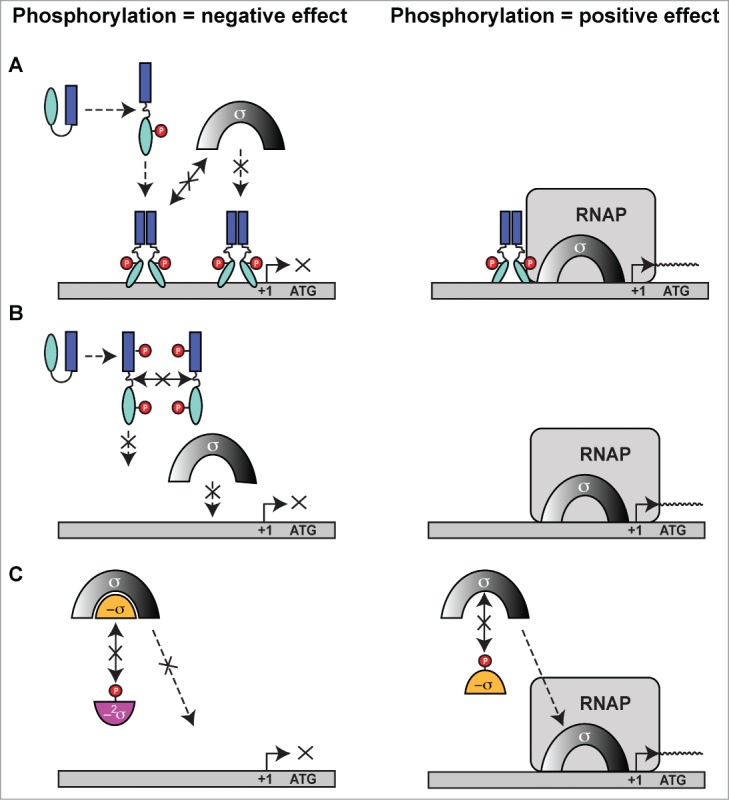
Possible mechanisms of eSTK/eSTP regulation of transcription. (**A**) eSTK phosphorylation of a transcription factor can cause either a negative effect (left) or positive effect (right) on the recruitment of transcriptional machinery through direct sigma factor and/or RNA polymerase (RNAP) interactions. (**B**) eSTK phosphorylation can also directly influence transcription factor binding to target DNA by either obstructing sister monomer interactions, or by directly affecting its physical hydrogen-bonding to target DNA. These influences can result in either a negative (left panel) or positive (right panel) effect on σ-factor/RNAP recruitment. (**C**) eSTK phosphorylation of both anti-sigma factors (-σ) and anti-anti-sigma factors (^−2^σ) can result in negative (left) or positive (right) effects on σ-factor/RNAP recruitment.

Bacterial eSTKs and eSTPs are ubiquitous signaling proteins found in virtually every important bacterial pathogen, where they are almost invariably required for infection and regulation of virulence. In recent years much progress has been made in understanding how these relays propagate pleiotropic phosphorylation to, for example, modify cell division, avoid host immunity and enable resistance to antimicrobials. One way these global modifications can be accomplished is by directly regulating transcription factors, and even core components of RNA polymerase to then, in turn, initiate a rewiring of transcriptional circuitry with even a single phospho-modification. An overview of possible mechanisms of eSTK/P control of transcription described thus far in the literature is summarized in **Fig. 10**. With the use of next generation sequencing, exciting possibilities now exist to understand the extent to which eSTK/P phosphorylation affects global transcriptional responses. In this regard, important future directions will include using such technology to determine a pathogen's transcriptome changes through eSTK/P signaling when variable tissues and environmental conditions are encountered during infection. With an ever-accumulating database of clinical isolates to sample, it will also be of future importance to determine how eSTK/Ps and their targets have co-evolved within the host, especially after treatments with antimicrobials. In support, dense genomic sampling of bacterial pathogens has revealed surprising changes in amino acid composition with the potential to accommodate new phosphorylatable sites, which could then enable efficient rewiring of infection and virulence traits. These discoveries combined with newly emerging prokaryotic PTMs such as tyrosine, cysteine and arginine phosphorylations will become increasingly important to more completely understand how the bacterial signaling network is used to successfully cause disease. Finally, a different yet equally important question to be answered is: How do eSTKs and eSTPs discriminate between a diverse array of cellular substrates to enable such precise and coordinated responses? Although specific substrate domain interactions such as FHA modules have been identified and explored, it remains to be determined how eSTK-substrate discrimination occurs with the rest of eSTK/P interacting partners. With the exception of SarA/MgrA family regulators and some sigma factor related proteins reviewed here, most modifications appear to occur on stable portions of the proteins with defined secondary and tertiary structures. Thus it is likely that to enable their substrate specificity, eSTKs and eSTPs might recognize a rigid 3-dimensional motif within their diverse *native* target substrate folds. For most eSTKs and eSTPs this has yet to be experimentally determined. To begin addressing this problem eSTK/P-substrate co-structural information and accompanying biochemical analyses must be produced. When combined with more randomized combinatorial approaches to substrate interactions, these data should help give us a more complete picture as to how these critical, and until recently underappreciated microbial signaling systems may be further exploited for design of novel antimicrobials.
